# Growth and welfare in mixed health system financing with physician dual practice in a developing economy: a case of Indonesia

**DOI:** 10.1007/s10754-020-09289-9

**Published:** 2020-11-07

**Authors:** Barış Alpaslan, King Yoong Lim, Yan Song

**Affiliations:** 1 Department of Economics, Social Sciences University of Ankara, Ankara, Turkey; 2 Centre for Applied Macroeconomic Analysis, ANU, Canberra, Australia; 3 Nottingham Business School, Nottingham Trent University, Nottingham, UK; 4 Institute of Politics and Economics, Nanjing Audit University, Nanjing, China

**Keywords:** Dual practice, Economic growth, Health care financing, Welfare, H51, I11, I15, O41

## Abstract

Based on Indonesia’s hybrid *BPJS Kesehatan* health system, we analyze for welfare-optimal government financing strategy in an economy with a mixed health system using an endogenous growth framework with physician dual practice. We find the model solution to produce two vastly different regimes in terms of policy implications: a “high” public-sector congestion regime as in the benchmark case of Indonesia, and a “low” public-sector congestion, high capacity regime. In the former, welfare-optimal health financing strategy appears to be promoting private health service. In contrast, in the low-congestion, high capacity regime, a welfare-optimal strategy is to do the opposite of increasing government physician wage at the expense of private health subsidy. These results highlight the importance of developing a benchmarking system that measures the actual degree of congestion faced by the public health service in a developing economy, as it ultimately would influence the optimal health financing strategy to be pursued.

**Electronic supplementary material:**

The online version of this article (10.1007/s10754-020-09289-9) contains supplementary material, which is available to authorized users.

## Introduction

The question on what constitutes the best policy approach in managing and financing a national health system in a developing economy remains a subject of much debate. As summarized in Saksena et al. (2010), for some the expansion of private health services to complement public health provision is desirable as this represents a gain in efficiency and the quality of health care, whereas for others the private sector is inherently inequitable and could create disincentive for health workers to provide their best effort in public practice, especially those who engage in dual practice (Berman and Cuizon 2004). Indeed, despite being prevalent in numerous developing-economy health system, physician dual practice can exist in various different forms due to the heterogeneity of national heath system across countries (McPake et al. 2011, 2014; Hort and Hipgrave 2013). In many developing economies, especially those with geographically sparse regions, the government often has to channel public funding to support private health facilities, in addition to the wage bill of government doctors. Indeed, the role of government in subsidizing private health service cost in developing economies is highlighted in Gina et al. (2012), who reviewed health insurance reforms in nine developing countries, and documented that many of these national health systems are “hybrid” in nature, i.e. private health care is also effectively public financed. This is epitomized by the “new” national health system of Indonesia post-2014, where true private voluntary health insurance is not well-developed and the “private” health financing schemes are government-owned, resulting in the government essentially supporting both public and private health care. In an attempt to achieve universal health coverage by 2019, Indonesia launched the *BPJS Kesehatan* system in 2014 and effectively doubled down on the government’s role in financing a mixed health system, since *BPJS* administers the national health insurance, *Jaminan Kesehatan Nasional*. This fascinating hybrid case of a national health system therefore provides an ideal case for our analysis of mixed health system financing.

Being the world’s fourth most populous country, Indonesia’s decentralized heath care system is an interesting case study for the evaluation of the macroeconomic effects of a mixed health financing system in which physician dual practice is common. Historically, out-of-pocket private health expenditure has played a more important role than public health spending in Indonesia. Despite this, private voluntary health insurance is not well developed in Indonesia, with the “private insurance” providers technically also government funded. For instance, of the three major health financing programs that existed pre-2014 reform into the *BPJS Kesehatan* system, the *P.T. Askes* program covers the civil servants and their dependents, the *Jamkesmas* is public funded to cover the poorest segment of the population, and *Jamsostek* is similar to a classic social insurance program for private sector employees managed by a state enterprise (Thabrany 2008).^1^ These historical institutional features therefore result in a hybrid mixed health system, with many of the features retained by the new national health insurance system, *BPJS Kesehatan*. In other words, in the conventional context of public (directly through government doctors’ wages) and private health financing (through private insurance), for Indonesia both of these financing elements are ultimately led back to the fiscal budget. Indeed, since its conception in 2014, the national system of *BPJS Kesehatan* has been facing a deficit, therefore providing a source of persistent pressure to the fiscal budget (Fossati 2017; Pisani et al. 2016). As seen later, these provide the main rationales for the design of our model.

The case of Indonesia raises important knowledge gaps in the present literature on health financing, which this study attempts to address. First, despite the large microeconomics literature focusing on examining the implications of physician dual practice and the welfare effects of different regulations, to our knowledge, the link to the overall financing of a national health system is not explored. Second, while there are existing theoretical growth models focusing on examining the links between health expenditure and economic growth, the effects of physician dual practice on consumers’ choice in a mixed health system have never been examined, especially in a developing economy context.^2^ Third, while there exists a rich variety of case studies and country-level reports, there remains a vacuum in terms of a theoretical study that seeks to identify the overall welfare implications associated with the dynamic trade-off of public and private health financing made by the government. We address these by developing an endogenous growth model with micro-foundations of a mixed health care system and physician dual-practice, to analyze for potential (or lack thereof) welfare-optimal government financing strategy for a mixed health system in developing countries. To preview, we find the model solution to produce two vastly different regimes in terms of policy implications: a “high” public-sector congestion regime as in our benchmark case of Indonesia, and a “low” public-sector congestion, high capacity regime. In the benchmark regime where public-sector capacity is low, we find that a government subsidy to private health care is both growth- and welfare-enhancing. This is more effective than a public-sector “rewarding” policy in raising government physicians’ wage if its goal is to improve physician effort in public practice. In this regime, welfare-optimal health financing strategy appears to be promoting private health service. In contrast, in the low-congestion, high capacity regime, a welfare-optimal strategy is to do the opposite of increasing government physician wage. Nevertheless, the private health subsidy policy is able to produce welfare-enhancing results in this regime too under certain scenarios. These findings suggest that the question of an optimal financing in Indonesia’s hybrid national health system does not have a straightforward answer, though the provision of private health subsidy is more likely to be welfare-enhancing if the true underlying regime cannot be ascertained. This may explain the current underperformance of Indonesia’s *BPJS Kesehatan* system, and the policy direction the government opted to go forward with by continuously reforming its private health care sector. These results also highlight the importance of developing a benchmarking system that measures the actual degree of congestion faced by the public health service, as it ultimately influences the optimal health financing strategy to be pursued.

The rest of the article is structured as follows. “Literature review” section provides a brief review on the relevant literature. “The model” section presents the model, and following the definition of the relevant equilibrium concepts, proceeds to solve for the dynamic system characterizing the model solutions. In “The model” section, the model is calibrated for Indonesia. After that, we analyze the model properties by implementing a series of policy experiments in “Model calibration and parametrization” section. “Policy experiments” section draws on policy implications from the experiments to conclude the article.

## Literature review

In the microeconomics literature on physician dual practice, it is well established that dual practice is prevalent in numerous health systems of developed and developing countries. For instance, in the UK approximately two-thirds of NHS (National Health Service) consultants have significant private work (Humphrey and Russell 2004). According to the newest 2016 Indonesian Family Life Survey, at least 50% of the public community physicians reported to have a private practice. Similarly, Gruen et al. (2002) believe that more than 80% of the government physicians in Bangladesh engage in private practice, while McPake et al. (2013) show that 55% of physicians surveyed in three African cities engaged in dual practice. The literature of dual practice in a mixed health market is limited but growing. Thorough reviews of existing literature of physician dual practice have been studied by Eggleston and Bir (2006), García-Prado and González (2007), and Socha and Bech (2011). Some studies, such as Ferrinho et al. (2004), Humphrey and Russell (2004), and Askildsen and Holmås (2013) explored the motivation of physician for having dual practice. The consensus arrived is that, public physicians engage in dual practice mainly due to the two reasons of financial incentive and strategic influence. These imply that, while low public pay does create the incentive for private practice (Ferrinho et al. 1998), physicians also appreciate the greater freedom and efficiency in the private sector. However, there are other studies that suggest that, allowing physician dual practice is costly and has negative impacts on the quality of public health care. González (2004, (2005) argue that dual-practice physician may over-provide medical services in public in order to increase his prestige, and divert low-cost patients to his private practice in order to achieve his financial aim. Brekke and Sørgard (2007) develop a theoretical model to argue that allowing physician dual practice will induce physicians to provide less supply or attention in the public sector, which in turn leads to lower overall health provision. However, they did suggest that allowing dual practice in a mixed health care market may be socially desirable. This view is shared by Biglaiser and Ct (2007), who develop another model that suggests allowing dual practice always enhances aggregate patients’ welfare, even though dual-practice physicians may refer patients to their private practices. Indeed, González and Macho-Stadler (2013) find that an outright ban on dual practice is seldom optimal, though the various scenarios they have examined suggest that different policy interventions may be warranted for different health systems.

As mentioned, while the microeconomics literature has reviewed the welfare effects of various regulations affecting dual practice, an explicit link to the relatively macroeconomic issue of government financing of the national health system is not well explored. By implication of this missing link that bridges the microeconomic and macroeconomic issues, the effect of dual practice on the average health status of the population and consequently, productivity and economic growth is also not well understood. This is despite over 200 years of empirical evidence on health-led growth discussed and documented in Madsen (2018). In the theoretical macro-literature, while the benefits of government’s health expenditure are well-documented (Chakraborty 2004; Agénor 2008, 2015), to our knowledge, the interaction between public and private expenditure on health has only been modeled in a simplistic manner. Specifically, in studies such as Bhattacharya and Qiao (2007), private health expenditure is modeled as generational investment for old age, while public expenditure as largely exogenous. Similarly, in studies such as Osang and Sarkar (2008) and Kunze (2014), the positive non-linearity between life expectancy and economic growth is explained in the context of inter-generational human capital investment. In essence, most of these studies, including peripheral models focusing on health-gender equality (Agénor et al. 2014; Agénor and Canuto 2015) and health-environment nexus (Mariani et al. 2010), model health mainly in the context of children, with the subsequent adult health status being a result of persistence. The feature of health-service quality that determines patients’ choice of health care and therefore directly on adult health status has therefore never been considered. Addressing this missing link between the microeconomics and macroeconomics of health literature is therefore our primary motivation.

## The model

Consider an economy with discrete time $t=0,1,...,\infty$. Population is constant and normalized to $\bar{N}=1$, and consists of two-period lived individuals (adulthood and old age; there is a constant $\alpha \in (0,1)$ share of adults in any given period *t*) with identical preferences, save for having different valuation of the quality of health services. Due to this difference, individuals’ *willingness-to-pay* for health care are mapped along a continuous distribution, indexed by $\xi \in (0,1)$. Individuals use *either* public health care (free of charge) or private health services. In each period, the consumption for health care arises due to an illness that occurs to each individual, and the illness is assumed to proportionately affect the health status of each individual with the same degree of severity.^3^ There is a survival probability of $\pi _{t}$ in which individuals survive to old age. Each individual is risk neutral and endowed with one unit of time in each period of life. In old age, time is allocated entirely to leisure. Savings are held only in the form of physical capital. Individuals have no other endowments, except for initial stocks of health ($h_{0}$) and physical capital ($K_{0}^{P}$) at time $t=0$.

In addition to private individuals, there is a representative physician who allocates his one unit of effort among leisure, public practice, and private practice. As in the theoretical literature on dual practice, such as Rickman and McGuire (1999), González (2005) and Brekke and Sørgard (2007), there is only one form of dual practice, and the latter is supplied to a price-taking private hospital. Given that the physician gets to set his *private-practice* wage, he will always prefer private practice. The effort in private practice is therefore demand-determined. The physician’s wage in the public health care system is paid by a balanced-budget government, who also spends on health infrastructure and subsidizes private health care cost incurred by the households. Lastly, there is a continuum of identical price-taking firms producing non-storable final goods used either for consumption or investment. There is imperfect information for firms in that they observe only the average labor efficiency level and therefore pays a common efficiency wage to all labor.^4^

### Preferences and health status

There is a continuum of individuals, mapped by $\xi \in (0,1)$, receives net wage income (paid by firms according to the average labor efficiency level in the economy) by supplying labor. The wage income is either saved, spent in consuming final goods, or for those who opt so, in private health care. Health services in the economy are provided either by public hospital (free of charge) or private hospital (incurring a health care cost, $hc_{t}$). $\xi$ follows a continuous distribution with density function $f(\xi )$ and cumulative distribution function $F(\xi )$. For tractability, $\xi$ is assumed to be uniformly distributed on its support, and enters additively into preferences of private health care users. As such, the expected lifetime utility at the beginning of period *t* of an individual $j=PH$, *GH* is given by

1 $$\begin{aligned} V_{t}^{j}=\left\{ \begin{array}{l} \ln (c_{t}^{j})^{\kappa }(h_{t}^{j})^{1-\kappa }+\varLambda \pi _{t}\mathbb {E} _{t}\left[ \ln (c_{t+1}^{j})^{\kappa }(h_{t+1}^{j})^{1-\kappa }\right] +\ln \xi _{t}, \\ \ln (c_{t}^{j})^{\kappa }(h_{t}^{j})^{1-\kappa }+\varLambda \pi _{t}\mathbb {E} _{t}\left[ \ln (c_{t+1}^{j})^{\kappa }(h_{t+1}^{j})^{1-\kappa }\right] \end{array} \begin{array}{l} \text {if }j=PH \\ \text {if }j=GH \end{array} ,\right. \end{aligned}$$

where $c_{t}^{j}$ ($c_{t+1}^{j}$) denotes consumption of final good in adulthood (old age) for individual *j*, $h_{t}^{j}$ ($h_{t+1}^{j}$) is the health status of an individual *j* in adulthood (old age), $\pi _{t}\in [0,1]$ is the survival probability common to all individuals regardless of whether they use private ($j=PH$) or public health care ($j=GH$), $\kappa$ measures the relative contribution of ordinary consumption to utility, $\varLambda <1$ is the discount factor, $\mathbb {E}_{t}$ the expectation operator conditional on information at time *t*. The specification assumes a realistic non-independence of ordinary consumption and health status, which is consistent with studies such as Agénor (2008). For simplicity, we assume individuals do not derive disutility from working.

The period-specific budget constraints are given by

2 $$\begin{aligned}&c_{t}^{j}+s_{t}^{j}=\left\{ \begin{array}{ll} (1-\tau )a_{t}^{A}w_{t}-hc_{t}, &{} \text {if }j=PH \\ (1-\tau )a_{t}^{A}w_{t} &{} \text {if }j=GH \end{array} ,\right. \end{aligned}$$

3 $$\begin{aligned}&\pi _{t}c_{t+1|t}^{j}=(1+r_{t+1})s_{t}^{j},\text { }j=PH,GH. \end{aligned}$$

where $a_{t}^{A}w_{t}$ is the efficiency wage ($a_{t}^{A}$ the average labor efficiency in the economy, $w_{t}$ the wage rate), $\tau \in (0,1)$ the tax rate, $s_{t}$ saving, and $r_{t+1}$ the rental rate of private capital in period $t+1$. Equation (3) indicates that individuals consume in old age ($t+1$) with a probability $\pi _{t}$, assumed as exogenous following Blackburn and Cipriani (2002).^5^ Solving for the individual’s utility maximization problem with respect to intertemporal consumption yields the Euler equation, $\mathbb {E}_{t}c_{t+1}^{j}/c_{t}^{j}=\varLambda (1+r_{t+1})$.

Due to the one-off nature of the illness and the heterogeneity in the *willingness-to-pay*, individuals’ choice of health services are solely determined by their relative position along the uniform distribution of $\xi$. In fact, as seen later, we can derive a threshold willingness-to-pay, $\xi _t^{\hat{c}}$, which would then allow for the determination of the share of patients using public health services, $\xi _{t}^{C}$, based on the cumulative distribution of $\xi$.

The actual health status of an individual $j=PH$, *GH* is therefore given by

4 $$\begin{aligned} h_{t}^{j}=\left\{ \begin{array}{l} \theta h_{0}(e_{t}^{PH})^{\nu _{H}}(H_{t}^{G})^{\nu _{C}}, \\ \frac{\theta h_{0}}{(\xi ^{C} _{t}\bar{N})^{\varkappa }}(e_{t}^{GH})^{\nu _{H}}(H_{t}^{G})^{\nu _{C}} \end{array} \begin{array}{l} \text {if }j=PH \\ \text {if }j=GH \end{array} ,\right. \end{aligned}$$

where $\nu _{H}$, $\nu _{C}\ge 0$, $\theta \in (0,1)$ measures the effect of the illness, $h_{0}$ is a constant baseline health status endowed to individuals at birth (and assumed to be same for all individual *j*), $e_{t}^{PH}$ and $e_{t}^{GH}$ the effort level allocated by the physician in private and public practice respectively, and $H_{t}^{G}$ the broad health infrastructure made available by the government for everyone in the economy. Unlike March and Schroyen (2005), we do not explicitly introduce a waiting time for public health service. Instead, the quality of public health service (and its impact on health status) is subject to congestion/capacity issue associated with the overall size of public patients, $\xi ^{C} _{t}\bar{N}$. Specifically, the more patients use free government health care (the larger $\xi ^{C} _{t}\bar{N}$ is), for a given (anti-)congestion parameter $\varkappa >0$, the effective congestion [denominator for $j=GH$ in (4)] will be larger, therefore lowering health status. Nevertheless, given that $\xi ^{C}_{t}\bar{N}\in [0,1]$, if we were interested instead in evaluating the impact of different congestion for a given public-sector patient size (as explored in “Extension and robustness” section), then the smaller the (anti-)congestion parameter value $\varkappa >0$ is, the larger the effectiveness congestion, $(\xi ^{C}_{t}\bar{N})^{\varkappa }$, will be.

For old age, given the presence of the non-zero mortality rate, we follow Agénor and Canuto (2015) to specify

5 $$\begin{aligned} h_{t+1}^{j}=\pi _{t}h_{t}^{j},\text { \ \ \ }j=PH,GH\text {.} \end{aligned}$$

Likewise, for simplicity, labor efficiency is specified as having a one-to-one relationship to health status, in that $a_{t}^{A}=h_{t}^{A},$ where $h_{t}^{A}=(1-\xi ^{C}_{t})h_{t}^{PH}+\xi ^{C}_{t}h_{t}^{GH}$, and let $\xi _{t}^{C}$ denote the share of individuals using public health services and $1-\xi _{t}^{C}$ the share using private. Equivalently, given (4),

6 $$\begin{aligned} h_{t}^{A}=\theta h_{0}(H_{t}^{G})^{\nu _{C}}\left[ (e_{t}^{PH})^{\nu _{H}}(1- \xi ^{C}_{t})+(e_{t}^{GH})^{\nu _{H}}\frac{(\xi ^{C}_{t})^{1-\varkappa }}{(\bar{N} )^{\varkappa }}\right] . \end{aligned}$$

An individual finds it optimal to pay for private health care if his/her expected lifetime utility exceeds the expected utility of using only public health care, $\mathbb {E}_{t}(V_{t}^{PH})\ge \mathbb {E}_{t}(V_{t}^{GH})$, or

7 $$\begin{aligned}&\ln (c_{t}^{PH})^{\kappa }(h_{t}^{PH})^{1-\kappa }+\varLambda \pi _{t}\mathbb {E}_{t}\left[ \ln (c_{t+1}^{PH})^{\kappa }(h_{t+1}^{PH})^{1-\kappa }\right] +\ln \xi _{t} \nonumber \\&\quad \ge \ln (c_{t}^{GH})^{\kappa }(h_{t}^{GH})^{1-\kappa }+\varLambda \pi _{t} \mathbb {E}_{t}\left[ \ln (c_{t+1}^{GH})^{\kappa }(h_{t+1}^{GH})^{1-\kappa } \right] . \end{aligned}$$

There exists a threshold value of the willingness-to-pay, $\xi _{t}^{\hat{c}}$, above which all individuals with higher value would opt to pay for private health care. In specifying (7), we assume that an individual knows if his/her willingness-to-pay is above or below the threshold $\xi _{t}^{\hat{c}}$ and can therefore decide whether to pay for private health care or not at the beginning of adulthood.^6^ For analytical tractability, the private (interpretable as out-of-pocket) health care cost is specified to be proportional to the gross efficiency wage income, where $hc_{t}=\mu _{t}a_{t}^{A}w_{t}$, with $\mu _{t}\in R$, $\mu _{t}$ $=(\mu _{0}-s_{t}^{H})$, $\mu _{0}$ $\in (0,1)$, and $s_{t}^{H}$ a private (per individual) health care subsidy provided by the government in period *t*.

As shown in Online Appendix, setting (7) as equality, we can derive a threshold value $\xi _{t}^{\hat{c}}$,

$$\begin{aligned} \xi _{t}^{\hat{c}}=\left[ \frac{1-\tau }{1-\tau -\mu _{t}}\right] ^{\kappa }\left( \frac{h_{t}^{PH}}{h_{t}^{GH}}\right) ^{\kappa -1}. \end{aligned}$$

Given that the share of individuals using public hospital equals $\xi _{t}^{C}=\bar{N}\xi _{0}^{C}\int _{0}^{\xi _{t}^{\hat{c}}}f(\xi )d\xi =\xi _{0}^{C}\xi _{t}^{\hat{c}}\bar{N}$ , and the share of individuals using private health service equals $1-\xi _{t}^{C}$, for some multiplicative constant $\xi _{0}^{C}\ge 0$, by using (4), we have:

$$\begin{aligned} \xi _{t}^{C}=\frac{\xi _{0}^{C}\varOmega _{t}}{(\bar{N})^{\frac{1+\varkappa (\kappa -1)}{1-\varkappa (1-\kappa )}}}\left( \frac{e_{t}^{PH}}{e_{t}^{GH}} \right) ^{\frac{\nu _{H}(\kappa -1)}{1-\varkappa (1-\kappa )}},\text { where } \varOmega _{t}=\left( \frac{1-\tau }{1-\tau -\mu _{t}}\right) ^{\frac{\kappa }{ 1-\varkappa (1-\kappa )}}, \end{aligned}$$

or equivalently, if $\bar{N}$ is normalized to one,

8 $$\begin{aligned} \xi _{t}^{C}=\xi _{0}^{C}\varOmega _{t}\left( \frac{e_{t}^{PH}}{e_{t}^{GH}} \right) ^{\frac{\nu _{H}(\kappa -1)}{1-\varkappa (1-\kappa )}},\text { where } \varOmega _{t}=\left( \frac{1-\tau }{1-\tau -\mu _{t}}\right) ^{\frac{\kappa }{ 1-\varkappa (1-\kappa )}}, \end{aligned}$$

which depends non-linearly on the ratio of physician effort between the two health care services.

### Private health care

There is a price-taking private hospital that receives its revenue in the form of the total private health care cost, $hc_{t}$, paid by the $1-\xi _{t}^{C}$ individuals. Taking this revenue, individuals’ health care choice, physician’s effort in public practice ($e_{t}^{GH}$), and the private-practice wage rate, $w_{t}^{PH}$ (determined monopolistic competitively by the physician) as given, the private hospital chooses the amount of physician effort, $e_{t}^{PH}$, to maximizes profits, $\max _{e_{t}^{PH}}\Pi _{t}^{PH}=(1-\xi _{t}^{C})\bar{N}\mu _{t}a_{t}^{A}w_{t}-w_{t}^{PH}e_{t}^{PH}$.

Given (6), and knowing that $\bar{N}=1$, we derive the demand function of $e_{t}^{PH}$,

9 $$\begin{aligned} e_{t}^{PH}=\left( \mu _{t}\nu _{H}\theta h_{0}\right) ^{\frac{1}{1-\nu _{H}} }\left( \frac{w_{t}}{w_{t}^{PH}}\right) ^{\frac{1}{1-\nu _{H}}}(H_{t}^{G})^{ \frac{\nu _{C}}{1-\nu _{H}}}(1-\xi _{t}^{C})^{\frac{2}{1-\nu _{H}}}. \end{aligned}$$

Even though there is only a single representative physician in the economy, the physician in dual practice ($e_{t}^{PH}>0$) will always prefer to meet the demand from private hospital, given that he/she has control of the private-practice wage rate. Given the perceived demand function, which determines the marginal revenue, the representative physician therefore behaves as if he/she is in a monopolistically competitive market by setting his/her “price”, $P_{t}^{PH}$, to maximize his/her payoff in the private sector. Given that the price in the perceived demand function is given in the price of $P_{t}^{PH}=w_{t}^{PH}/w_{t}$, the physician maximizes $\Pi _{t}^{I}=(P_{t}^{PH}-1)e_{t}^{PH}$. Using (9), we derive the optimal private-practice “price” to be a constant mark-up of:

10 $$\begin{aligned} P_{t}^{PH}=\frac{w_{t}^{PH}}{w_{t}}=\frac{1}{\nu _{H}}. \end{aligned}$$

### Public health care

The optimal level of $e_{t}^{GH}$ is derived by evaluating the physician’s participation condition in dual practice. The effort level supplied by the physician for public practice, $e_{t}^{GH}$, can be determined using a simple specification developed in Agénor and Aizenman (1999). Specifically, the representative physician has one unit of effort, which is to be spent among leisure, public practice, and private practice. In each period *t*, the physician evaluates a period utility function that depends on the wage and effort in both practices, $U^{D}[w_{t}^{PH},w_{t}^{GH},e_{t}^{PH},e_{t}^{GH}]$. Without losing any generality, a log-utility specification means, for the physician to involve in dual practice, the utility derived from dual practice has to be at least as good as the utility derived from solely practicing in the public health care system:

11 $$\begin{aligned}&\ln \left\{ [e_{t}^{GH}w_{t}^{GH}+e_{t}^{PH}w_{t}^{PH}]^{ \delta _{R}}(1-e_{t}^{PH}-e_{t}^{GH})^{1-\delta _{R}}\right\} \nonumber \\&\quad \ge \ln [(e_{t}^{GH}w_{t}^{GH})^{\delta _{R}}(1-e_{t}^{GH})^{1-\delta _{R}}] \text {, where }\delta _{R}\in (0,1). \end{aligned}$$

In the margin, the physician is indifferent between dual practice and solely practice in the public sector. Setting (11) as equality, we solve for an expression of the optimal public-practice effort level:

12 $$\begin{aligned} e_{t}^{GH}=\frac{(1-e_{t}^{PH})\left( 1+\frac{e_{t}^{PH}w_{t}^{PH}}{ e_{t}^{GH}w_{t}^{GH}}\right) ^{\psi }-1}{\left( 1+\frac{e_{t}^{PH}w_{t}^{PH}}{ e_{t}^{GH}w_{t}^{GH}}\right) ^{\psi }-1},\text { \ where \ }\psi =\delta _{R}/(1-\delta _{R}). \end{aligned}$$

As such, instead of specifying dual practice as having a negative effect on public performance, as in one of the scenarios examined in González and Macho-Stadler (2013), public effort, $e_{t}^{GH}$, in this framework is a direct outcome from physician’s optimizing behavior. In each period *t*, the total income of physician, $e_{t}^{PH}w_{t}^{PH}+e_{t}^{GH}w_{t}^{GH}$, is subject to the same tax rate, $\tau$. For simplicity, the physician is assumed to spend all the after-tax income and does not save.

### Firms

There is a continuum of identical firms, indexed by *i* $\in (0,1)$, producing non-storable final goods used either for consumption or investment. Production requires the use of effective labor and private capital rented from households. Assuming a Cobb-Douglas technology, the production function of firm *i* takes the form,

13 $$\begin{aligned} Y_{t}^{i}=A_{0}\left[ \frac{K_{t}^{P}}{(K_{t}^{P,i})^{\varsigma }}\right] ^{\omega }(a_{t}^{A}N_{t}^{i})^{\beta }(K_{t}^{P,i})^{1-\beta }, \end{aligned}$$

where $A_{0}\ge 0$, $a_{t}^{A}$ denotes the average, economy-wide labor efficiency (which is the same for all firms), $K_{t}^{P,i}$ the firm-specific stock of capital, $K_{t}^{P}=\int _{0}^{1}K_{t}^{P,i}di$ the aggregate private capital stock, $N_{t}^{i}$ the number of adult workers employed by firm *i*, $\varsigma ,\omega \ge 0$, and $\beta \in (0,1)$. While production exhibits constant returns to scale in firm-specific inputs, similar to Lim (2017), production also benefits from an Arrow-Romer type of learning externality associated with the economy-wide aggregate private capital stock, which is subject to a congestion effect of $\varsigma$.

The inputs’ markets are competitive, and firms do not observe the actual health status of individuals. As such, for labor market equilibrium to hold, law of one price ensures that there can only be one wage rate in the economy, $w_{t}$. All firms therefore observe the wage rate and pay $w_{t}$. Each firm’s profit maximization problem is given by $\max _{N_{t}^{i},K_{t}^{P,i}}\Pi _{t}^{i}=Y_{t}^{i}-r_{t}K_{t}^{P,i}-w_{t}a_{t}^{A}N_{t}^{i}$. Given that all firms are identical in symmetric equilibrium, $a_{t}^{A}=h_{t}^{A}$, let $\alpha \bar{N}$ be the total number of adults in the population, where $\alpha \in (0,1)$, in a symmetric equilibrium we have the first-order conditions:

14 $$\begin{aligned} w_{t}=\beta Y_{t}/(h_{t}^{A}\alpha \bar{N}),\text { \ \ \ }r_{t}=(1-\beta )Y_{t}/K_{t}^{P}. \end{aligned}$$

### Government

The government taxes effective wages of both the adult individuals and the representative physician at a constant rate $\tau$. It spends a total of $G_{t}^{H}$ on medical research and broad infrastructure, $G_{t}^{G}$ on physician’s wage in public practice, $G_{t}^{S}$ on a private health care subsidy, and $G_{t}^{U}$ on other (unproductive) items. It cannot issue bonds and must therefore run a balanced budget in any period *t*, where

15 $$\begin{aligned} G_{t}^{H}+G_{t}^{G}+G_{t}^{S}+G_{t}^{U}=\tau \alpha \bar{N} a_{t}^{A}w_{t}+\tau (e_{t}^{PH}w_{t}^{PH}+e_{t}^{GH}w_{t}^{GH}). \end{aligned}$$

Shares of spending are constant fractions of revenues:

16 $$\begin{aligned} G_{t}^{h}=\upsilon _{h}\left[ \tau \alpha \bar{N}a_{t}^{A}w_{t}+\tau (e_{t}^{PH}w_{t}^{PH}+e_{t}^{GH}w_{t}^{GH})\right] ,\text { \ \ \ }h=H,G,S,U, \end{aligned}$$

where $\upsilon _{h}\in (0,1)$, and $\sum \upsilon _{h}=1$. This specification is consistent with studies such as Chakraborty (2004) and Agénor (2015).

We know that the total bill of private health care subsidy adds up to $G_{t}^{S}=s_{t}^{H}a_{t}^{A}w_{t}\alpha \bar{N}(1-\xi _{t}^{C})$, which when equating to (16), means the per-individual health care subsidy provided by the government is:

17 $$\begin{aligned} s_{t}^{H}=\frac{\upsilon _{S}\tau }{(1-\xi _{t}^{C})}\left[ \frac{e_{t}^{PH}w_{t}^{PH}+e_{t}^{GH}w_{t}^{GH}}{a_{t}^{A}w_{t}\alpha \bar{N}}+1\right] . \end{aligned}$$

Also, given that the total wage bill for public practice is given by $G_{t}^{G}=e_{t}^{GH}w_{t}^{GH}$, we have

18 $$\begin{aligned} e_{t}^{GH}w_{t}^{GH}=\frac{\tau \upsilon _{G}}{1-\tau \upsilon _{G}}\left[ \alpha \bar{N}a_{t}^{A}w_{t}+e_{t}^{PH}w_{t}^{PH}\right] . \end{aligned}$$

To account for both the learning effect from the improving aggregate labor efficiency level in the economy ($a_{t}^{A}\bar{N}$), the production of health infrastructure is modeled as

19 $$\begin{aligned} H_{t}^{G}=H_{0}^{G}[\frac{a_{t}^{A}\alpha \bar{N}}{(K_{t}^{P,i})^{\varsigma }}]^{\eta }(\varphi G_{t}^{H})^{\epsilon }, \end{aligned}$$

where for consistency, the learning externality $\eta \ge 0$ is specified to be subject to the same congestion factor as in the private sector, and $\varphi ,\epsilon \in (0,1)$ capture the spending efficiency.

## Model solution

First, we assume that the representative physician spends all the net after-tax income he/she earns in each period *t*. The asset market-clearing condition therefore only requires the private capital stock in period $t+1$ to be equal to the aggregate savings made by adults in period *t*. Assuming full depreciation (a reasonable assumption for a long-range model in which individuals live for two periods), we have

20 $$\begin{aligned} K_{t+1}^{P}=\alpha \bar{N}s_{t}=\alpha \bar{N}[(1-\xi _{t}^{C})s_{t}^{PH}+\xi _{t}^{C}s_{t}^{GH}], \end{aligned}$$

where $\alpha \in (0,1)$ is the share of adults in the population in each period *t*.

With the saving-investment balance in equilibrium, note that it is trivial to show that the other markets clear. In a closed economy with balanced-budget government, the amounts that are neither consumed nor taxed (which is used to pay for government expenditure) are fully reflected in savings. By definition, equation (20) therefore reflects the final goods market equilibrium too.

### Definition 1

A competitive equilibrium for this economy is characterized by the sequences $\{c_{t},c_{t+1},s_{t}\}_{t=0}^{\infty }$ by individuals, effort by the general physician $\{e_{t} ^{PH},e_{t}^{GH}\}_{t=0}^{\infty }$, private capital stock $\{K_{t+1}^{P}\}_{t=0}^{\infty }$, prices $\{w_{t},w_{t}^{GH} ,w_{t}^{PH},r_{t+1}\}_{t=0}^{\infty }$, health status $\{h_{t}^{GH},h_{t}^{PH},h_{t}^{A},h_{t+1}^{A}\}_{t=0}^{\infty }$, private health care subsidy $\{s_{t}^{H}\}_{t=0}^{\infty }$, as well as the resulting sequences of the endogenous share of individuals using public health care $\{\xi _{t}^{C}\}_{t=0}^{\infty }$, such that, for a given set of constant policy parameters $\tau ,\upsilon _{H}$,$\upsilon _{G},\upsilon _{S},\upsilon _{U}$, and initial stocks $K_{0}^{P}$, $h_{0},H_{0}^{G}>0$, individuals maximize utility, representative physician maximizes payoff, firms maximize profits, the product and asset markets clear, and the government budget is balanced.

### Definition 2

A balanced growth equilibrium (BGE) is a competitive equilibrium in which (i) $c_{t}$, $c_{t+1}$, $Y_{t}$, and $K_{t}^{P}$ all grow at the constant rate $\gamma$; (ii) all health status ($h_{t}^{GH}$, $h_{t} ^{PH}$, $h_{t}^{A}$) are constant; (iii) by implications of (i)-(ii), the wage rates ($w_{t},w_{t}^{GH},w_{t}^{PH}$) grow at the same rate as $Y_{t}$; and (iv) the private capital rental rate and private health care subsidy ($r_{t}$, $s_{t}^{H}$) are constant. In the BGE, the equilibrium share of individuals using public health care ($\xi _{t}^{C}$) is therefore constant too.

The wage-growth and heath status-constancy characteristics in the BGE (instead of having it as wage being constant and health status growing like capital) are consistent with empirical evidence documented in Hartwig (2008, (2010), where health expenditure is mainly driven by wage growth over time, with limited growth-enhancing evidence from health capital formation. Next, to generate endogenous growth, given that $K_{t}^{P,i}=K_{t}^{P}\forall i$ in the symmetric equilibrium, we impose the theoretical

### Assumption

$\omega (1-\varsigma )=\beta$, $\eta \varsigma =\epsilon$, which would then turn (13) into the standard Y-K form of $\frac{Y_{t}}{K_{t}^{P}}=A_{0}(h_{t}^{A})^{\beta }$. In Online Appendix, the expressions for the growth rate of private capital stock and final output are also derived, with the steady-state growth rate given by

21 $$\begin{aligned} 1+\gamma =(\tilde{h}^{A})^{\beta }\tilde{\sigma }\beta [(1-\tilde{\xi } ^{C})(1-\tau -\tilde{\mu })+\tilde{\xi }^{C}(1-\tau )], \end{aligned}$$

where $\tilde{h}^{A}$, $\tilde{H}^{G}$, $\tilde{\sigma }$, $\tilde{\xi }^{C}$, and $\tilde{\mu }$ are the steady-state values of the respective variables. Further, to study the transition dynamics of the model, from Online Appendix, we derive a non-linear dynamic equation of the average health status, $h_{t+1}^{A}=f(h_{t}^{A})$:

22 $$\begin{aligned} h_{t+1}^{A}=\frac{\pi _{t}\theta h_{0}\alpha ^{(\eta -\epsilon )\nu _{C}-\varkappa }\left( 1+\frac{e_{t}^{PH}}{\nu _{H}}\right) ^{\epsilon \nu _{C}}}{(\varphi \beta \upsilon _{H}\tau \varPhi _{2})^{-\epsilon \nu _{C}}}\left[ \begin{array}{c} (e_{t}^{PH})^{\nu _{H}}(1-\xi _{t}) \\ +(e_{t}^{GH})^{\nu _{H}}(\xi _{t})^{1-\varkappa } \end{array} \right] (h_{t}^{A})^{[\eta -(1-\beta )\epsilon ]\nu _{C}}, \end{aligned}$$

where $$\varPhi _{2}=\varphi \beta \upsilon _{H}\tau \varPhi _{1}$$, $$\varPhi _{1}=1+[\tau \upsilon _{G}/(1-\tau \upsilon _{G})]$$.

Given that capital and output growth in this economy is financed by savings, which in turn is a function of two main factors: (1) productivity, assumed to have a one-to-one mapping with average health status; (2) distribution of public health users and private health users, the non-linear first-difference equation, therefore Eq. (22) serves as the key dynamic equation driving economic growth. In Eq. (22), along the transition path, growth depends non-linearly on existing health status, fiscal policy parameters such as the spending shares ($\upsilon _{H}$ and $\upsilon _{G}$ directly enter into the equation, albeit non-linearly, whereas the private health subsidy share, $\upsilon _{S}$, has an indirect effect through its influence on the private practice effort and the individuals’ consumption-saving decisions), as well as a weighted combination of the chosen physician effort in both private and public health care services. Given that this is a general equilibrium model, both the equilibrium effort levels, as well as the shares of patients choosing public versus private health care, are mutually dependent and endogenous to each other. This means comparative static analysis with respect to these variables would yield analytically ambiguous results and depend on the configuration of the different parameter values. As such, numerical analysis-based policy experiments will be required.

Given this, as well as the complexity of the system, stability of the economy cannot be studied analytically. However, it is established numerically by first solving for both an initial BGE and end-period BGE [with a change in policy parameter(s)], then deriving the transitional paths using finite-difference methods similar to Fair and Taylor (1983), Trimborn et al. (2008).

## Model calibration and parametrization

To examine the overall implications of dual practice, as well as the growth effects of public and private health care financing, we calibrate the model to Indonesia, a Southeast Asian developing economy whose health care financing system reform towards achieving universal coverage in the previous decade has been well-documented (Rokx et al. 2009; Fossati 2017; González et al. 2017; Pisani et al. 2016). Further, the public availability of micro-data from 5 waves of *Indonesia Family Life Survey* (IFLS 1–5) conducted by RAND researchers (see, for instance, Strauss et al. 2004, 2009, 2016) allows some model parameters in model equations to be empirically estimated, which we believe vastly improves reliability of findings from numerical policy experiments. In addition, macro-data from the Global Health Observatory, World Health Organization (WHO) and World Bank’s World Development Indicators databases are used. In sum, our calibration strategy focuses on matching the initial steady-state values (denoted with tilde) to the first statistical moment of relevant variables, so as to achieve a baseline that stylistically illustrates Indonesia during the period 2000–15. The parameters and initial steady-state values of variables summarized in Tables 1 and 2.

**Table 1:** Parameter values: benchmark case

Parameter	Description	Value
*Households*		
$\varLambda$	Discount factor	0.907
$\kappa$	Relative contribution to utility	0.342
$\nu _{H}$	Elasticity (health) wrt physician effort	0.072
$\nu _{C}$	Elasticity (health) wrt infrastructure	0.55
${ \theta }$	Parameter, inverse of effect of illness	0.56
$\alpha$	Shares of adults in the population	0.68
*Health system and Physician*		
$\varkappa$	(Anti-)congestion parameter, public health care	0.5
$\mu _{0}$	Time-invariant private health care cost	0.109
$\delta _{R}$	Relative physician preference, wage income	0.524
*Firms*		
$\omega$	Elasticity, Arrow-Romer externality	0.184
$\beta$	Elasticity wrt effective labor	0.67
*Public sector*		
${ \tau }$	Tax rate on wages	0.171
$\upsilon _{G}$	Share of spending on physician wage	0.004
$\upsilon _{H}$	Share of spending on health infrastructure	0.0486
$\upsilon _{S}$	Share of spending on private health subsidy	0.022
$\eta$	Public infrastructure, learning externality	0.30
$\varphi$	Parameter, spending efficiency	0.368
$\epsilon$	Elasticity wrt spending flow, stock	0.55

**Table 2:** Initial steady-state values of key variables

Variable	Description	Value
$\tilde{\xi }^{C}$	Share of individuals using public health care	0.828
$\tilde{e}^{PH}$	Effort allocated to private practice, physician	0.344
$\tilde{e}^{GH}$	Effort allocated to public practice, physician	0.299
$\tilde{h}^{A}$	Average adult health status	100.0
$\tilde{H}^{G}$	Stock of health infrastructure	1.000
$\tilde{\mu }$	Private health care cost, fraction of market wage	0.086
$\tilde{s}^{H}$	Private health care subsidy rate	0.023
$\tilde{w}$	Market wage rate	1.000
$\tilde{w}^{PH}$	Physician wage rate, private practice	13.889
$\tilde{w}^{GH}$	Physician wage rate, public practice	0.938
$\tilde{\pi }$	Adult survival probability	0.734
$\gamma$	Final output growth rate	0.053

First, for households, following Blackburn and Cipriani (2002), we treat adult survival rate as exogenous and set $\tilde{\pi }=$ 0.734, based on WHO’s mortality data for the 30–70 years-old age group in 2015.^7^ Next, for the discount factor, $\varLambda$, we parameterize it based on Havranek et al. (2015) meta-analysis of the elasticity of intertemporal substitution for Indonesia, and yields $\varLambda =1/1.102=0.907$. Given this, knowing that the steady-state savings rate is given by $\tilde{\sigma }=\tilde{\pi }\varLambda /(1+\tilde{\pi }\varLambda )$, yields $\tilde{\sigma }=0.40$. This is a high value but within reasonable range of Indonesia’s recent gross savings rates that are in excess of 30%. Further, from the Online Appendix, we also know that $\varLambda =(1+\gamma )/(1+\tilde{r})$ must hold in the BGE. Given that the average real GDP growth rate of Indonesia, $\gamma =0.053$ during 2000–15, this means the steady-state rental rate of physical capital, $\tilde{r}=0.161$, which is within average business lending rates in Indonesia. The relative contribution of ordinary consumption to utility, $\kappa$, is parameterized based on the relative preference parameter values for consumption and health in Agénor and Canuto (2015), which yields $\kappa =0.342$. The elasticity parameter of health status with respect to health service infrastructure, $\nu _{C}=0.55$ is set based on Osang and Sarkar (2008), while the elasticity with respect to physician effort, $\nu _{H}$, is empirically estimated using the IFLS micro-data based on the log-linearized form of (10).^8^ The estimated intercept term suggests a mark-up value of approximately 13.9 times, which yields $\nu _{H}=1/13.9=0.072$. This suggests a benchmark of highly inelastic health response with respect to physician effort. Given the critical role of this parameter in determining the effects of dual practice, extensive sensitivity analysis is further implemented later. For the parameter measuring the effect of illness prior to treatment, $\theta$, given that this is non-directly observable in practice, we set $\theta =0.56<\tilde{\pi }$, which is proxied by the UHC services coverage index of 56% for Indonesia, published by the WHO.

Next, on the physician and health system parameters, the relative preference of physician towards wage income, $\delta _{R}$, is empirically estimated based on a log-linear approximated form of the marginal condition, (12). More specifically, by exploring the cross-sectional properties of the responses in the IFLS, for the sample of Puskesmas Head with dual-practice, we proxy $e_{t}^{GH}$ with the (patient-adjusted) working hours per week by the Puskesmas head in the government health centre, and regress it against the ratio of the Head’s total income from joint-practice (revenue plus basic salary) over his/her basic salary as Puskesmas Head, which yields an estimated coefficient of 0.095, or equivalently, $\psi =\exp (0.097)=1.100$. This then allows us to derive $\delta _{R}=0.524$. For the private health care cost, from the WHO data in 2000–15, the average out-of-pocket and domestic private health expenditure (as percentage of health expenditure) are 49.25% and 64.27% respectively. Further, by dividing both out-of-pocket and domestic private health expenditure per capita (PPP $\$178.4$ and PPP $\$226$) by the surveyed mean income per capita (PPP $\$2,073.2$) in 2015, we get 0.086 and 0.109 respectively. The former gives $\tilde{\mu }=0.086$, while the latter $\mu _{0}=0.109$. By implications, the steady-state private health care subsidy rate is $\tilde{s}^{H}=0.023$. Given the absence of comparable estimates in the literature and the relevant variable in the IFLS dataset, the (inverse) capacity congestion parameter associated with the size of patients seeking free public health care, $\varkappa$, is set at 0.5, with its effects further examined using sensitivity analysis later. Lastly, the share of adults in the total population, $\alpha =0.68$, is set, in line with the percentage of working-age population (ages 15–64) in Indonesia.

For the production parameters, the elasticity value of Arrow-Romer externality, $\omega$, is set at 0.184, in line with meta-analysis of Bom and Ligthart (2014). The output elasticity to labor, $\beta$, is set to the national accounting-based empirical estimate for Indonesia, which gives $\beta =0.67$ (Aswicahyono et al. 2013). For the government, the effective tax rate, ${\small \tau }$, is calibrated as follows. Tax revenue as percentage of GDP averages at 0.114 in the period 2000–15. Given a labor income share of 0.67, we calculate $\tau =0.114/0.67=0.171$. On the spending shares, the share of government spending on private health subsidy can be computed by dividing the difference between domestic private and out-of-pocket health expenditure by the general government expenditure, yielding an average of $\upsilon _{S}=0.022$ for the period 2000-15. By similar logic, we know the total domestic general government health expenditure as percentage of general government expenditure averages at 0.0526, which is the sum of $\upsilon _{G}$ and $\upsilon _{H}$. We further calculate the value of $\upsilon _{G}$ by first, estimating the total wage bill for government doctors. Specifically, based on the physician density of 0.292 per 1000 people, we estimated the total number of physicians and then multiplied it with the sample mean-annual basic salary of Puskesmas Head reported in IFLS-5. After that, we divide the estimated wage bill by the general government expenditure in current prices, yielding $\upsilon _{G}=0.004$. Given this, we then parametrize $\upsilon _{H}=0.0526-0.004=0.0486$. For the production of health infrastructure, the learning externality parameter, $\eta$, is parametrized using the value of Alpaslan and Ali (2018), which equals 0.3. The country-specific government spending efficiency parameter for Indonesia is calculated based on the index values of Dabla-Norris et al. (2012), $\varphi =1.47/4.0=0.368$. Lastly, following Agénor et al. (2014), the elasticity of health infrastructure with respect to spending flow is set at $\epsilon =0.55$. The parameter values of benchmark are summarized in Table 1.

The remainders of the benchmark steady-state values of key variables in the BGE are determined as follows, and summarized in Table 2. First, we decide to normalize the initial steady-state value of $$\tilde{H}^{G}$$ and $$\tilde{w}$$ to 1.0, while setting health status as an index of 100. These are obtained by adjusting the multiplicative terms, $$h_{0}$$, $$H_{0}^{G}$$, and $$A_{0}$$. Second, based on responses in the IFLS Survey, 53.5% of Puskesmas Head engage in dual practice while 46.5% practice solely in public health services. Assuming that the responses revealed the preference of physicians in allocating their effort, we therefore have $$\tilde{e}^{GH}/$$ $$\tilde{e}^{PH}=0.868$$. From the survey, the average hours spent by a government physician in Puskesmas are 35.87, which given 5-working days, implies $$\tilde{e}^{GH}=35.87/(5$$*$$24)=0.299$$. Given these, we calculate $$\tilde{e}^{PH}=0.344$$.^9^ Third, we determine the steady-state values to be set for the three wage rates. From the survey, the average basic salary of a Puskesmas Head is used as a proxy for $$\tilde{w}^{GH}$$. The difference between this and the average reported monthly earnings allows us to then determine $$\tilde{w}^{PH}$$. Given the empirically estimated value for $$\nu _{H}$$ and the normalization of $$\tilde{w}$$ to unity, we know from (10) that $$\tilde{w}^{PH}=13.889$$, which in turn allows us to determine $$\tilde{w}^{GH}=0.938$$. Fourth, to determine the steady-state shares of individuals using public and private health care, we utilize the steady-state version of (17), and yields $$1-\tilde{\xi }^{C}=0.172$$. This means $$\tilde{\xi }^{C}=0.828$$.

## Policy experiments

To study the model properties, we first consider two individual policy experiments, which involve the government reallocating 0.01 share of its budget from non-productive spending to finance: (i) an increase in government spending on public physicians’ wage ($\upsilon _{G}$ increases from 0.004 to 0.014); and (ii) an increase in government’s subsidy to private health care ($\upsilon _{S}$ from 0.022 to 0.032).

**Fig. 1:**
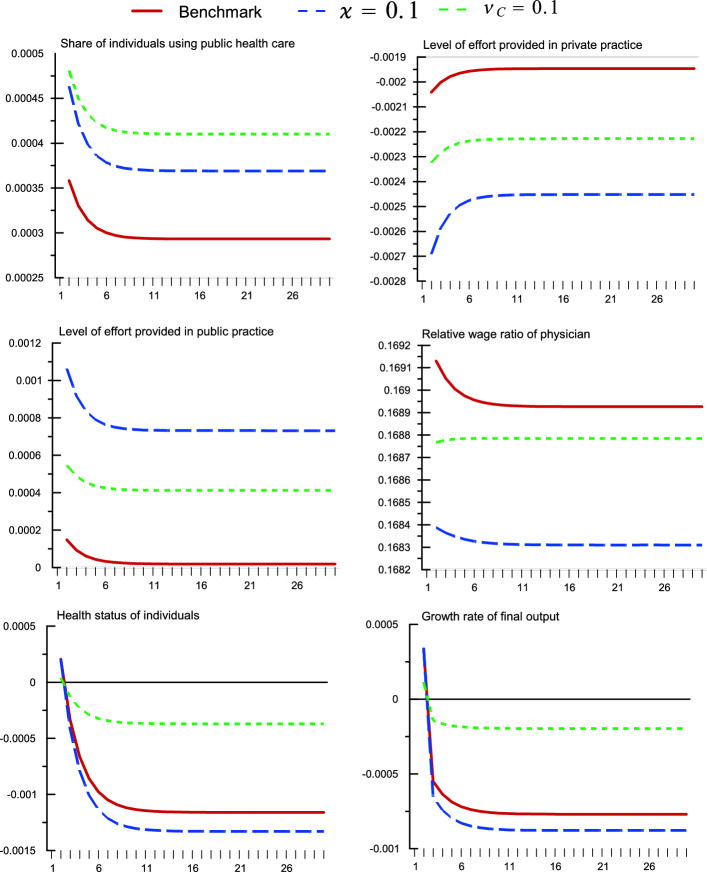
A permanent increase in government physician wage rate. An increase in ($\upsilon _{G}$) from 0.004 to 0.014 (Percentage deviation for health status; Absolute deviations for others)

**Fig. 2:**
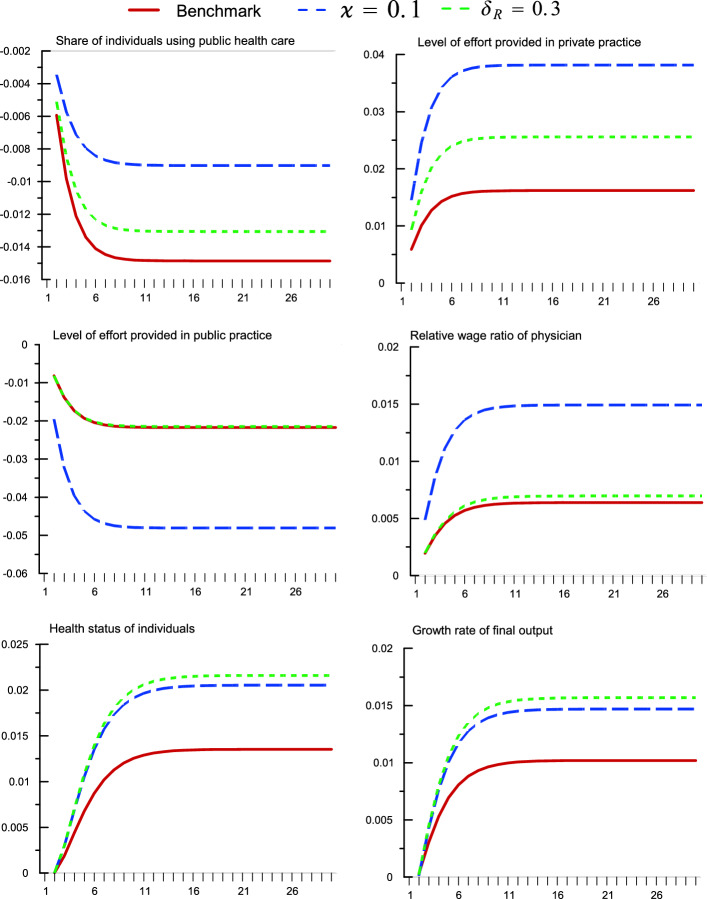
A permanent increase in government spending on private health subsidy. An increase in ($\upsilon _{S}$) from 0.022 to 0.032 (Percentage deviation for health status; Absolute deviations for others)

To measure the permanent effects on welfare, given that our economy consists of two-period lived individuals, we adopt the perfect-foresight welfare criterion of models with similar structure, such as Agénor and Lim (2018). Specifically, assume now that the economy consists of infinite number of generations of two-period lived individuals that will replenish by nature, the social welfare function is then given by a discounted sum of utility along the BGE path across an infinite sequence of individuals (De la Croix and Philippe 2002), as in $\mathcal {W}_{t}=\displaystyle \sum \limits _{h=0}^{\infty }\varPsi ^{h}[\tilde{\xi }_{0}^{C}V_{t+h}^{GH}+(1-\tilde{\xi }_{0}^{C})V_{t+h}^{PH}]$, where $\varPsi$ $\in (0,1)$ is the social discount factor, $V_{t+h}^{GH}$ and $V_{t+h}^{PH}$ measure the indirect utility functions of individuals using public and private health services respectively, $\tilde{\xi }_{0}^{C}$ and $1-\tilde{\xi }_{0}^{C}$ are time-invariant constant weights, which we set to equal the initial steady-state shares of households using public and private health care respectively.^10^ For simplicity, we restrict the social welfare function to only measure those of the private individuals. Also for tractability, we restrict our analysis to welfare along the BGE path, with an approximation derived in Online Appendix. All shocks considered are permanent in nature, with the steady-state effects of key variables summarized in Table 3 and the relevant transition dynamics in Figs. 1, 2.^11^ In addition to benchmark results, Table 3 also presents selected sensitivity analysis results.

**Table 3:** Results summary for policy experiments: steady-state effects (Percentage deviations for health status and social welfare; absolute deviations from baseline for all others)

	Initial values	Benchmark	$\varkappa = 0.1$	$\varkappa = 5.0$	$\nu _H= 0.7$	$\nu _C= 0.1$	$\kappa = 0.8$	$\delta _R= 0.3$
An increase in government spending on public physicians’ wage ($\upsilon _G$)								
Share of public patients	0.828	0.0003	0.0004	0.0001	0.0002	0.0004	− 0.0004	− 0.0042
Relative effort ratio (public/private)	0.868	0.0050	0.0084	− 0.0040	0.0003	0.0069	− 0.0243	− 0.0637
Physician’s effort level in public practice	0.299	0.0000	0.0007	− 0.0019	− 0.0047	0.0004	− 0.0062	0.0019
Physician’s effort level in private practice	0.344	− 0.0019	− 0.0025	− 0.0006	− 0.0055	− 0.0022	0.0026	0.0296
Ratio of public-practice wage rate to private-practice wage rate	0.068	0.1689	0.1683	0.1706	1.5840	0.1688	0.1747	0.1747
Public-practice wage rate	0.938	2.3475	2.3391	2.3699	2.2766	2.3446	2.4250	2.3970
Private-practice wage rate	13.889	0.0053	0.0061	0.0038	0.0062	0.0017	− 0.0066	− 0.1193
Health status*	100.0	− 0.0012	− 0.0013	0.0008	− 0.0130	− 0.0004	0.0014	0.0265
Growth rate	0.053	− 0.0008	− 0.0009	0.0006	− 0.0092	− 0.0002	0.0010	0.0180
Social welfare*	29.6	− 0.0010	− 0.0012	0.0006	− 0.0147	− 0.0003	0.0365	0.0230
An increase in government spending on private health subsidy ($\upsilon _S$)								
Share of public patients	0.828	− 0.0117	− 0.0149	− 0.0035	− 0.0091	− 0.0133	− 0.0076	− 0.0131
Relative effort ratio (public/private)	0.868	− 0.0992	− 0.2121	0.3002	− 0.0062	− 0.1215	0.0829	− 0.1180
Physician’s effort level in public practice	0.299	− 0.0217	− 0.0481	0.0596	− 0.0013	− 0.0268	0.0173	− 0.0215
Physician’s effort level in private practice	0.344	0.0162	0.0382	− 0.0375	0.0009	0.0202	− 0.0118	0.0256
Ratio of public-practice wage rate to private-practice wage rate	0.068	0.0064	0.0149	− 0.0115	0.0046	0.0071	− 0.0040	0.0070
Public-practice wage rate	0.938	0.0843	0.1997	− 0.1599	0.0057	0.0973	− 0.0542	0.0896
Private-practice wage rate	13.889	− 0.0615	− 0.0929	− 0.0006	− 0.0013	− 0.0155	0.0124	− 0.0977
Health status*	100.0	0.0135	0.0205	0.0001	0.0028	0.0034	− 0.0027	0.0216
Growth rate	0.053	0.0102	0.0147	0.0019	0.0029	0.0028	− 0.0007	0.0157
Social welfare*	29.6	0.0133	0.0197	− 0.0001	0.0047	0.0042	− 0.0075	0.0203

Both health status and social welfare are in index, and therefore is calculated as percentage deviation from initial baseline value

Both experiments involve the government making an additional 0.01 share of its expenditure to the specific category, by reducing non-productive expenditure

*Source*: Authors’ calculations

**Table 4:** An increase in government physicians’ wage, for different value of $\varkappa$ and $\nu _{H}^{*}$ (Percentage deviation from initial baseline level of social welfare)

$\varkappa$		$\nu _{H}$	
0.1	− 0.0012	0.10	− 0.0012
0.5	− 0.0010	0.20	− 0.0024
1.0	− 0.0009	0.30	− 0.0040
1.5	− 0.0019	0.40	− 0.0060
2.0	− 0.0173	0.50	− 0.0085
2.012	− 0.0242	0.60	− 0.0114
$$\text {Structural break}^{**}$$		0.70	− 0.0147
		0.80	− 0.0185
		0.90	− 0.0229
3.412	0.0422	1.10	− 0.0334
3.5	0.0149	1.20	− 0.0397
4.0	0.0059	1.30	− 0.0469
4.5	0.0029	1.40	− 0.0550
5.0	0.0006	1.50	− 0.0643

*This involves an increase in $\upsilon _{G}$ from 0.004 to 0.014

**Model is not solvable between the range of $\varkappa$ = 2.012–3.412

### An increase in government physician wage rate

Consider a one percentage-point permanent increase in government allocation to public physicians’ wage ($\upsilon _{G}$). In Fig. 1, we see that this instantaneously increases the public-practice wage rate, therefore raises the relative wage ratio. For a given level of effort, it becomes optimal for the physician to have a higher relative ratio of (public-private) practice effort. While the relative effort ratio increases, in the margin of (12) and at the initial level of demand for private-practice effort, this means that there is a substitution effect driving down the required level of effort in public practice. On the opposite end, the direct income effect brought about by the increase in public physicians’ wage puts upward pressure to public physician effort. In combination, the net effect is positive to the level of effort provided in public practice.

At the same time, from (8), an increase in the relative (public-private) effort level means an improvement in public health service quality, which leads to an increase in the share of individuals using public health care. On impact, the overall effects on average health status and therefore growth rate of final output are positive. However, in the long-run as the economy moves towards the new steady state, the fall in the demand for private health care leads to lower total revenue of the private hospital, which in turn leads to a decline in demand for the effort in private practice. A combined decrease in effort level by the physician—albeit gradually—leads to a gradual decline in the total tax income of the government. Given that government’s investment in broad health infrastructure and private health subsidy is a constant fraction of the total tax income, this means lower stock of infrastructure and higher effective (out-of-pocket) household expenditure on health. These negate the initial positive effect on average health status and leads to a gradual decline in effective market wage received by average workers. This reduces the income of private individuals and consequently, consumption. In the steady state, these various general equilibrium mechanisms translate to mildly negative effect on health status ($-0.12$%), growth ($-0.08$%), and welfare ($-0.10$%). These suggest that, in a mixed health system where dual practice is prevalent and the private sector plays a significant role in the overall landscape, the fall-out associated with the general equilibrium effects from an increase in government physician wage rate can be negative, despite its positive effect on public physicians’ effort level. Indeed, this appears to be robust across most of the sensitivity analysis considered in Table 3, save for when the public sector has a very high capacity/experiences very low congestion ($\varkappa =5.0$), utility/preference specific with very low contribution from health status ($\kappa =0.8$), or when the physician places low weight on her/his total earnings ($\delta _{R}=0.3$). In these three cases, the opposite of a positive steady-state effect on average health status, growth, and welfare are observed. Relating these to the literature, despite a vastly different theoretical approach, the findings are in consistent with the recommendation documented in García-Prado and González (2011), where “rewarding” policies for public-sector physicians are only recommended for more developed economies (which are likely to have higher capacity in public health provision). However, the dynamic trade-off in growth (between instantaneous and steady-state effects) found is new to the literature.

### An increase in spending on private health subsidy

Next, consider a one percentage-point permanent increase in government allocation to private health subsidy ($\upsilon _{S}$). This can be interpreted as a stylized representation of the government increasing its support on private health care, as illustrated by Indonesia’s *BPJS Kesehatan* system in 2014. Unlike the $\upsilon _{G}$ shock, we see in Fig. 2 that the policy effects of key variables are consistent for both instantaneous and in the long-run steady state, with the transition dynamics behaving largely monotonically. On impact, an increase in $\upsilon _{S}$ raises the per-individual private health subsidy provided by the government, which reduces the total out-of-pocket health care cost of the private individuals. More individuals are then willing to pay for private health care, resulting in an increase in the share of individuals using private health service, $1-\xi _{t}^{C}$. Both the increase in the total private health expenditure and private individuals using private health service mean the private hospital responds by demanding more private practices (in terms of effort) from the physician. This increases the effort level supplied to private practice, at a smaller trade-off of a drop in public-practice effort level, therefore a net expansion in total physician effort. The average health status therefore increases. In relative terms, the relative public-private effort ratio declines. The effective reduction in private health care cost also translates to higher consumption and savings, the latter results in an increase in the rate of physical capital investment and consequently, final output growth. Overall in the long run, average health status in the steady state increases by 1.3%, final output growth by 1.0%, and social welfare by 1.3%. Intuitively, given that the model specification is such that private effort is market demand-driven, a policy that reduces effective out-of-pocket expenditure on health is necessarily growth- and welfare-enhancing. Analytically, it is more interesting to also examine the sensitivity analysis scenarios. In all the non-$\varkappa$ scenarios, the results are largely consistent with the benchmark results, though the welfare decreases in the case when there is a larger (lower) relative contribution of goods consumption (health status) to the utility of households. The parameter $\varkappa$, which measures the (inverse) congestion factor of the public health service, seems to be critical in determining the structural behaviors of the model. While it is intuitive that a greater spending on private health care in a national health system with plenty of slack in its public-sector health care ($\varkappa =5.0$) is growth- and welfare-deteriorating,^12^ which is along the line of arguments made in studies such as Berman and Cuizon (2004), the steady-state growth and welfare effects can be slightly negative when public-sector health service congestion is set to a very high level ($\varkappa =0.1$). Although the effects of this ‘two-regime’ characteristic is not as obvious as in the previous experiment, this seems to suggest a non-monotonic model response with regards to the parameter $\varkappa$. Further examinations are therefore implemented later using sensitivity analysis.

### Further analysis: financing of a mixed health system

Having examined the benchmark experiments, we investigate the model further by analyzing the two measures for different values of $\varkappa$ and $\nu _{H}$. The critical role of the former is suggested in the benchmark experiments, while the latter directly influences the optimal mark-up of private practice, as seen in (10). Specifically, by focusing on the welfare effects, we repeated the two experiments across the continuous range of values for the two parameters, with results at selected intervals presented in Tables 4 and 5. From the two tables, our consideration in “An increase in government physician wage rate” section is confirmed numerically, that the solution system would experience a structural break in a certain range of $\varkappa$, hence creating a two-regime solution. On one hand, in the low public-sector congestion, high $\varkappa$ regime, the welfare effect of an increase in government physicians’ wage is actually positive, though the more slack the public health care system has (larger $\varkappa$), the lower the welfare gains. On the other hand, in the “normal” regime (where the benchmark case is in), the welfare effect is negative, as discussed in “An increase in government physician wage rate” section. However, the more congested the public health care system is (such as longer waiting lines, poor emergency response rates), the lower the welfare loss is associated with a pay rise for government physicians.

Similar two-regime solution is observed when the policy of an increase in private health subsidy is examined across the range of $\varkappa$. In the low public-sector congestion, high $\varkappa$ regime, the welfare effect of greater private health financing by the government is negative, though the negativity is smaller the more slack the public health care system has. In the normal regime, an increase in private health subsidy is mostly welfare-enhancing, though at extreme cases where the public sector is experiencing significant capacity issue, further support provided to private health service can be welfare-deteriorating to the overall economy.

In contrast to $\varkappa$, the solution system appears to behave largely monotonic across different private-practice wage mark-ups, $1/\nu _{H}$. For a “rewarding” scheme that raises government physicians’ wage, with the benchmark value of $\varkappa =0.5$, the negative welfare effect is smaller the higher the degree of mark-up charged by the physician in private practice. In contrast, for the experiment of an increase in private health subsidy, positive welfare effects are consistently observed across all domains considered for the parameter, $\nu _{H}$.

**Table 5:** An increase in private health subsidy, for different value of $\varkappa$ and $\nu _{H}^{*}$ (Percentage deviation from initial baseline level of social welfare)

$\varkappa$		$\nu _{H}$	
0.1	0.0197	0.10	0.0133
0.5	0.0133	0.20	0.0052
1.0	0.0098	0.30	0.0045
1.5	0.0055	0.40	0.0043
2.0	0.0064	0.50	0.0044
2.012	0.0103	0.60	0.0045
$$\text {Structural break}^{**}$$		0.70	0.0047
		0.80	0.0049
		0.90	0.0052
3.412	− 0.0212	1.10	0.0060
3.5	− 0.0085	1.20	0.0064
4.0	− 0.0032	1.30	0.0069
4.5	− 0.0010	1.40	0.0074
5.0	− 0.0001	1.50	0.0080

*This involves an increase in $\upsilon _{S}$ from 0.022 to 0.032

**Model system not solvable between the range of $\varkappa$ = 2.012–3.412

**Table 6:** Trade-offs between a change in government physicians’ wage versus a change in private health subsidy, for different congestion parameters (Percentage deviation from the initial baseline level of social welfare)

$$\varkappa = \mathbf{0}.5$$										
		$$\upsilon _G$$								
		− 0.003	− 0.002	− 0.001	0.000	+ 0.001	+ 0.002	+ 0.003	+ 0.004	+ 0.005
$\upsilon _S$	**− 0.003**	− 0.00311	− 0.00326	− 0.00340	− 0.00352	− 0.00364	− 0.00376	− 0.00387	− 0.00397	− 0.00408
	**− 0.002**	− 0.00196	− 0.00211	− 0.00225	− 0.00237	− 0.00249	− 0.00260	− 0.00272	− 0.00282	− 0.00292
	**− 0.001**	− 0.00079	− 0.00094	− 0.00107	− 0.00108	− 0.00132	− 0.00143	− 0.00154	− 0.00168	− 0.00178
	**0.000**	0.00041	0.00026	0.00013	**0.00000**	− 0.00012	− 0.00023	− 0.00034	− 0.00045	− 0.00055
	**+ 0.001**	0.00163	0.00148	0.00135	0.00122	0.00110	0.00099	0.00088	0.00078	0.00068
	**+ 0.002**	0.00288	0.00273	0.00260	0.00247	0.00235	0.00224	0.00213	0.00202	0.00193
	**+ 0.003**	**0.00415**	0.00400	0.00387	0.00374	0.00362	0.00352	0.00341	0.00330	0.00320

$$\varkappa =\mathbf{5.0}$$										
		$$\upsilon _G$$								
		− 0.003	− 0.002	− 0.001	0.000	+ 0.001	+ 0.002	+ 0.003	+ 0.004	+ 0.005
$\upsilon _S$	**− 0.003**	0.00022	0.00026	0.00029	0.00032	0.00036	0.00040	0.00045	0.00052	**0.00063**
	**− 0.002**	0.00012	0.00015	0.00018	0.00021	0.00026	0.00030	0.00036	0.00042	0.00050
	**− 0.001**	− 0.00006	0.00007	0.00008	0.00011	0.00016	0.00021	0.00029	0.00034	0.00041
	**0.000**	− 0.00005	− 0.00002	0.00000	**0.00000**	0.00007	0.00012	0.00015	0.00020	0.00035
	**+ 0.001**	− 0.00003	− 0.00002	− 0.00002	− 0.00001	− 0.00001	− 0.00001	− 0.00001	− 0.00001	0.00000
	**+ 0.002**	− 0.00005	− 0.00004	− 0.00003	− 0.00003	− 0.00002	− 0.00002	− 0.00002	− 0.00001	− 0.00001
	**+ 0.003**	− 0.00009	− 0.00007	− 0.00005	− 0.00004	− 0.00003	− 0.00003	− 0.00002	− 0.00001	− 0.00001

Next, Table 6 presents the results associated with an examination of a direct trade-off in health financing. Specifically, by assuming that the government faces a limitation in its ability to reallocate funds away from non-productive expenditure, it must choose between public or private health financing where an increase in one category must be financed by the other. Starting from the benchmark combination of $\upsilon _{G}=0.004$ and $\upsilon _{S}=0.022$, we examine this trade-off under the two different regimes of $\varkappa =0.5$ and $\varkappa =5.0$. In a normal regime (represented by the benchmark case, $\varkappa =0.5$), welfare-optimal health financing strategy appears to be promoting private health service, even if it is at the expense of public-sector physician wages. In contrast, in a low-congestion, high capacity regime ($\varkappa =5.0$), a welfare-optimal strategy is to do the opposite of increasing government physician wage at the expense of private health subsidy.

The various examinations suggest that the question of an optimal financing of a mixed health system in developing countries does not have a straightforward answer. The different capacity or congestion issue faced by public-sector health care can result in two different regimes. Further, due to the presence of a dynamic trade-off observed in “An increase in government physician wage rate” section, the policy window would matter too. Perhaps, these would partly explain the current underperformance of Indonesia’s *BPJS Kesehatan* system. Theoretically, while these findings are relatively new in the macroeconomics of health literature, similar two-regime results have been documented in microeconomic models such as McPake et al. (2007), which examined the flow of cross-subsidy between superior and basic service in a two-tier charging system, despite a completely different theoretical approach.

### Extension and robustness

To further evaluate the robustness of the results, notably the dual-regime characteristics identified, we modify the model by relaxing two assumptions: (i) survival probability is common to all individuals; and (ii) (anti-)congestion parameter is constant. Specifically, on the former, the survival probability that appears in (3), (5) is endogenized to be a function of health status, hence depending on the health service chosen in the previous period, as in:

23 $$\begin{aligned} \pi _{t}^{j}=\left\{ \begin{array}{l} \pi _{0}^{PH}(\frac{h_{t}^{PH}}{h_{0}})^{\phi _{PH}}, \\ \pi _{0}^{GH}(\frac{h_{t}^{GH}}{h_{0}})^{\phi _{GH}}, \end{array} \begin{array}{l} \text {if }j=PH \\ \text {if }j=GH \end{array} ,\right. \end{aligned}$$

where $\pi _{0}^{PH},\pi _{0}^{GH}\ge 0$ are initial time-invariant probability values, $h_{0}$ constant initial health [as in (4)], $\phi _{PH},\phi _{GH}\ge 0$ are the elasticity parameters of survival probability with respect to the heath status post-treatment by private and public health care respectively. Given this, saving rate is different between the two groups, and the aggregate health status in the economy is then just a weighted average of the health status of public health care users and private health care users:

24 $$\begin{aligned} h_{t}^{A}=\tilde{\xi }_{t}^{C}h_{t}^{GH}+(1-\tilde{\xi }_{t}^{C})h_{t}^{PH}. \end{aligned}$$

We solve this modified version of the model, and then simulate the two policy experiments of a one percentage-point permanent increase in government allocation to public physicians’ wage ($\upsilon _{G}$) and a one percentage-point permanent increase in government allocation to private health subsidy ($\upsilon _{S}$) again. The results are compared to those from benchmark analysis in Table 7. As seen for most variables, even though the probability is endogenized and therefore different between the two groups, the difference in policy effects are minimal (up to 5 decimal places). Qualitatively, the general equilibrium effects on economic growth and social welfare are similar.

In addition to $\varkappa =0.5$ (same value as benchmark model, hence same regime of “ high” public-sector congestion), we also examine the two policies for when $\varkappa =5.0$, which belongs to the “ low” public-sector congestion, high capacity regime. The novel finding of a dual regime from Tables 4, 5, 6 remains robust. As such, even when survival probability is different between individuals seeking public and private health care, we find that a government subsidy to private health care is preferable in the “ high” public-sector congestion regime; in the low-congestion, high capacity regime, a welfare-optimal strategy is instead to do the opposite of increasing government physician wage.

For the second extension, we endogenize the (anti-)congestion parameter, $\varkappa$, to be a function of the share of population using public health care. Specifically, we use a simple function of:

25 $$\begin{aligned} \varkappa _{t}=\varkappa _{0}(\tilde{\xi }_{t}^{C})^{-\phi _{\varkappa }}, \end{aligned}$$

where $\varkappa _{0}\ge 0$, $\phi _{\varkappa }>0$ is the elasticity of the (anti-)congestion parameter with respect to the size of public patients. Mathematically, this means the larger the share of population using public service, the smaller $\varkappa _{t}$ become, which then resulting in the *effective* congestion brought about by a given public patient size [denominator for $j=GH$ in (4)] will be larger. This then translates a lower post-treatment health status for the public health care users. In other words, an additional ‘aggravation’ effect associated with public health care is introduced.

Table 8 presents the results for the two policy experiments considered: one set for the case when the initial value of $\varkappa _{t}$ is set to 0.5, whereas in another the initial value of $\varkappa _{t}$ is set to 5.0. Overall, we have results that are consistent with the dual-regime finding when the policy of an increase in government physician wage rate is considered. However, for the policy of an increase in private health subsidy, the low public-sector congestion regime ($\varkappa =5.0$) would now produce positive welfare effects too. This appears to suggest that, a government subsidy to private health care is likely to be more welfare-enhancing in Indonesia, whereas for the policy of increasing public sector wage rate, it is only welfare-enhancing in the low-congestion, high capacity regime.

**Table 7:** Policy experiments: endogenizing survival probability to different health status (Percentage deviations for health status and social welfare; Absolute deviations from baseline for all others)

	Initial values	Benchmark (when $\pi$ is fixed)	$\varkappa = 0.5$	$\varkappa = 5.0$
An increase in government spending on public physicians’ wage ($\upsilon _G$)		Endogenous $\pi$		
Share of public patients	0.828	0.0003	0.0003	0.0001
Relative effort ratio (public/private)	0.868	0.0050	0.0050	− 0.0040
Physician’s effort level in public practice	0.299	0.0000	0.0000	− 0.0019
Physician’s effort level in private practice	0.344	− 0.0019	− 0.0019	− 0.0006
Ratio of public-practice wage rate to private-practice wage rate	0.068	0.1689	0.1689	0.1706
Public-practice wage rate	0.938	2.3475	2.3474	2.3699
Private-practice wage rate	13.889	0.0053	0.0054	0.0037
Health status*	100.0	− 0.0012	− 0.0012	0.0008
Growth rate	0.053	− 0.0008	− 0.0009	0.0006
Social welfare*	29.6	− 0.0010	− 0.0011	0.0006
An increase in government spending on private health subsidy ($\upsilon _S$)		Endogenous $\pi$		
Share of public patients	0.828	− 0.0117	− 0.0117	− 0.0035
Relative effort ratio (public/private)	0.868	− 0.0992	− 0.0990	0.3005
Physician’s effort level in public practice	0.299	− 0.0217	− 0.0217	0.0597
Physician’s effort level in private practice	0.344	0.0162	0.0162	− 0.0375
Ratio of public-practice wage rate to private-practice wage rate	0.068	0.0064	0.0065	− 0.0116
Public-practice wage rate	0.938	0.0843	0.0853	− 0.1609
Private-practice wage rate	13.889	− 0.0615	− 0.0709	0.0082
Health status*	100.0	0.0135	0.0156	0.0018
Growth rate	0.053	0.0102	0.0126	0.0004
Social welfare*	29.6	0.0133	0.0160	− 0.0013

Aggregate health status is computed as a weighted average of health status of public and private patients. Both health and social welfare are in index and therefore is calculated as percentage deviation from initial baseline value

Both experiments involve the government making an additional 0.01 share of its expenditure to the specific category, by reducing non-productive expenditure

*Source*: Authors’ calculations

**Table 8:** Policy experiment results: endogenizing the (anti-)congestion parameters; Comparing between the 2 Regimes with different initial congestion (Percentage deviations for health status and social welfare; absolute deviations from baseline for all others)

	Low $\varkappa$ regime		High $\varkappa$ regime	
	Initial values	Deviation	Initial values	Deviation
An increase in government spending on public physicians’ wage ($\upsilon _G$)				
Share of public patients	0.828	0.0003	0.828	0.0000
Relative effort ratio (public/private)	0.868	0.0065	0.868	− 0.0040
Physician’s effort level in public practice	0.299	0.0003	0.299	− 0.0019
Physician’s effort level in private practice	0.344	− 0.0022	0.344	− 0.0006
Ratio of public-practice wage rate to private-practice wage rate	0.068	0.1684	0.068	0.1700
Public-practice wage rate	0.938	2.3412	0.938	2.3644
Private-practice wage rate	13.889	0.0111	13.889	0.0156
Health status*	100.0	− 0.0024	100.0	0.0034
Growth rate	0.053	− 0.0016	0.053	0.0024
Social welfare*	29.6	− 0.0019	29.6	0.0021
(Anti-)congestion parameter	0.500	− 0.0019	5.000	0.0135
An increase in government spending on private health subsidy ($\upsilon _S$)				
Share of public patients	0.828	− 0.0123	0.828	− 0.0028
Relative effort ratio (public/private)	0.868	− 0.1339	0.868	0.3030
Physician’s effort level in public practice	0.299	− 0.0297	0.299	0.0601
Physician’s effort level in private practice	0.344	0.0225	0.344	− 0.0378
Ratio of public-practice wage rate to private-practice wage rate	0.068	0.0124	0.068	− 0.0058
Public-practice wage rate	0.938	0.1494	0.938	− 0.1095
Private-practice wage rate	13.889	− 0.2933	13.889	− 0.4580
Health status*	100.0	0.0668	100.0	0.1070
Growth rate	0.053	0.0472	0.053	0.0076
Social welfare*	29.6	0.0493	29.6	0.0059
(Anti-)congestion parameter	0.500	0.0809	5.000	0.0175

Both health status and social welfare are in index, and therefore is calculated as percentage deviation from initial baseline value

Both experiments involve the government making an additional 0.01 share of its expenditure to the specific category, by reducing non-productive expenditure

*Source*: Authors’ calculations

## Concluding remarks

We develop an endogenous growth model with a mixed health care system and physician dual-practice, to analyze the growth and welfare effects associated with different financing choice made by the government of a developing country. The model is calibrated illustratively for Indonesia, which has a well-documented hybrid *BPJS Kesehatan* health system. The main implications of this study were summarized in the introduction and need not be repeated here. We therefore conclude by pointing out that the model could be extended with more elaborative micro-structures, such as features that allows for the examination of two-tier pricing strategy (McPake et al. 2007), and other health policies and regulation issues evaluated in García-Prado and González (2007). Similarly, the modeling of the preference of physician has also been vastly simplified as a self-contained measure. For future extension, the supply of health workers in the economy can be fully endogenized to be modeled as driven by skills acquisition decision in the economy. On the empirical front, given the lack of country-level time series data on physician practice, pay rates, and patients’ choice of health services beyond those of IFLS 1–5 means a comprehensive empirical modeling of the theoretical framework developed in this article cannot be examined. This presents an obvious future research avenue that is worth exploring.

## Electronic supplementary material

Below is the link to the electronic supplementary material.

Supplementary material 2 (tex 28 KB)

## Footnotes

1 Also, see Rokx et al. (2009) for further description of the major health financing programs in Indonesia prior to the introduction of the current *BPJS Kesehatan* system. In short, despite being structured as private institutions that also sell private commercial policies, *P.T. Askes* and *P.T. Jamsostek* were administered by the Ministry of Health and were therefore akin to private health service provider being subsidized by the government. While both have been succeeded by *BPJS Kesehatan* and *BPJS Ketenagakerjaan* under the new system, similar structures of government insurance companies are retained.

2 It is well-documented empirically that the causal effects between health expenditure and economic growth are significantly different between developed and developing economies. For instance, see Rana et al. (2019). The policy implications derived on mixed practice from the existing theoretical contributions, primarily focusing on developed economies, are therefore non-transferable to a developing economy context.

3 This assumption is commonly made in general equilibrium model to ensure that in the steady state, symmetry allows for analytically tractable solution system to be derived. In practice, the degree of severity faced by each individual after he/she has fallen ill will be different, but some degree of homogeneity or aggregation is usually assumed. Indeed, Biglaiser and Ct (2007) and Brekke and Sørgard (2007) are the examples of theoretical studies that make simplification assumption that is similar to ours.

4 Overall, these model specifications are consistent with the key features of the hybrid health system in Indonesia (or broadly developing Southeast Asia), as described in studies such as Rokx et al. (2009), Fossati (2017). However, two deviations from reality are of note and are made for analytical simplification: (i) although small (in 2010, the number of physicians per 1000 people in Indonesia is only 0.14), there are obviously more than one physician in Indonesia; (ii) Indonesia has consistently maintained a small budget deficit in the range of 0.7–2.5% over the past 10 years. To model these two features will involve introducing framework of endogenous skills acquisition and debt dynamics, which are peripheral to our objectives but come with the cost of greater model complexity.

5 This specification follows Agénor (2015) and essentially assumes the presence of an actuarially fair annuity market that channels savings to investment in physical capital for the production in the next period. With the annuity market, old-age survivors share the savings plus interest left by savers who die in adulthood. The effective rate of return to saving would thus be $(1+r_{t+1})/\pi _{t}$, which would yield an equation similar to (3). Alternatively, it can also be assumed that the saving left by individuals who fail to survive to old age is confiscated by the government, who then spends it for unproductive purposes.

6 Equation (7) is assumed to hold as a strict inequality for the individual with the highest $\xi$, that is, $\xi =1$, otherwise nobody would choose to pay for private health care.

7 Endogenous mortality is a topic that is well examined in the macroeconomics literature on health and growth, notably in studies such as Osang and Sarkar (2008). However, it is peripheral to the core issues examined in this article. The introduction of endogenous survival rate, for instance to average health status, makes little difference to the results beyond amplifying the magnitudes of response by variables to policy shocks experimented (due to the reinforcing effect of a change in average health status). It is therefore abbreviated from this article.

8 Specifically, by matching the surveyed wage data from households and the monthly revenue data from community survey of Puskesmas Heads based on province and team code of the field team, we run different variations of a log-log regression of the monthly revenue (in excess of basic salary) of doctors with Private Practice on the wage income of households, controlling for factors such as community centre, province, and household IDs.

9 These imply that the number of hours spent in private practice is 41.28. The sum of private and public hours is therefore approximately 77 h in 5 days, or 15 h per week. This phenomenon of overstretched physicians and long working hours is well documented in Indonesia and more broadly, developing Southeast Asia.

10 Alternatively, weights based on steady-state relative shares (which deviate from baseline values as a result from each experiment), could be used. Qualitatively the results are broadly similar to those reported. Given that we focus only on the welfare change between the 2 steady states (and not along the transition), the “constant shares” specification is retained.

11 Similar to Agénor and Lim (2018) and other models examining transitional dynamics in the literature, there is a distinction between generational periods (*T*) and simulated period (*t*). In principle, *T* corresponds to 30 years in a two-period lived structure, as reflected in the assumption of full depreciation of physical capital. However, all of the other parameters and variables either do not have a time dimension or are calibrated on the basis of average annual data. For the numerical experiments, the intended length of a unit of time interval is therefore $t=1/30$, or best understood as one year.

12 Given that an extremely large magnitude of $\varkappa =5.0$ is set for this specific sensitivity scenario, the large negative absolute deviations observed for growth and social welfare should therefore be interpreted with caution. Indeed, the same disclaimer should be made for other results generated, in that the absolute magnitude observed is purely in an experimental context.

## References

[CR1] Agénor PR (2008). Health and infrastructure in a model of endogenous growth. Journal of Macroeconomics.

[CR2] Agénor PR (2015). Public capital, health persistence and poverty traps. Journal of Economics.

[CR3] Agénor PR, Aizenman J (1999). Macroeconomic adjustment with segmented labor markets. Journal of Development Economics.

[CR4] Agénor PR, Canuto O (2015). Gender equality and economic growth in brazil: A long-run analysis. Journal of Macroeconomics.

[CR5] Agénor PR, Lim KY (2018). Unemployment, growth and welfare effects of labor market reforms. Journal of Macroeconomics.

[CR6] Agénor PR, Canuto O, da Silva LP (2014). On gender and growth: The role of intergenerational health externalities and women’s occupational constraints. Structural Change and Economic Dynamics.

[CR7] Alpaslan B, Ali A (2018). The spillover effects of innovative ideas on human capital. Review of Development Economics.

[CR8] Askildsen JE, Holmås TH (2013). Wages and work conditions as determinants for physicians’ work decisions. Applied Economics.

[CR9] Aswicahyono H, Hill H, Najoko D (2013). Pathways to industrialization in the twenty-first century: New challenges and emerging paradigms: Indonesian industrialization: A latecomer adjusting to crises. Oxford Scholarship.

[CR10] Berman, P., & Cuizon, D. (2004). Multiple public-private job-holding of health care providers in developing countries: An exploration of theory and evidence. Issue paper Department for International Development Health Systems Resource Centre.

[CR11] Bhattacharya J, Qiao X (2007). Public and private expenditures on health in a growth model. Journal of Economic Dynamics and Control.

[CR12] Biglaiser G, Ct Albert Ma (2007). Moonlighting: Public service and private practice. The RAND Journal of Economics.

[CR13] Blackburn K, Cipriani GP (2002). A model of longevity, fertility and growth. Journal of Economic Dynamics and Control.

[CR14] Bom PR, Ligthart JE (2014). What have we learned from three decades of research on the productivity of public capital?. Journal of Economic Surveys.

[CR15] Brekke KR, Sørgard L (2007). Public versus private health care in a national health service. Health Economics.

[CR16] Chakraborty S (2004). Endogenous lifetime and economic growth. Journal of Economic Theory.

[CR17] De la Croix D, Philippe M (2002). A theory of economic growth: Dynamics and policy in overlapping generations.

[CR18] Dabla-Norris E, Brumby J, Kyobe A, Mills Z, Papageorgiou C (2012). Investing in public investment: An index of public investment efficiency. Journal of Economic Growth.

[CR19] Eggleston K, Bir A (2006). Physician dual practice. Health Policy.

[CR20] Fair RC, Taylor JB (1983). Solution and maximum likelihood estimation of dynamic nonlinear rational expectations models. Econometrica.

[CR21] Ferrinho P, Lerberghe WV, Julien MR, Fresta E, Gomes A, Dias F, Gonçalves A, Bäckström B (1998). How and why public sector doctors engage in private practice in portuguese-speaking African countries. Health Policy and Planning.

[CR22] Ferrinho P, Van Lerberghe W, Fronteira I, Hipólito F, Biscaia A (2004). Dual practice in the health sector: Review of the evidence. Human Resources for Health.

[CR23] Fossati D (2017). From periphery to centre: Local government and the emergence of universal healthcare in indonesia. Contemporary Southeast Asia A Journal of International and Strategic Affairs.

[CR24] García-Prado A, González P (2007). Policy and regulatory responses to dual practice in the health sector. Health Policy.

[CR25] García-Prado A, González P (2011). Whom do physicians work for? An analysis of dual practice in the health sector. Journal of Health Politics, Policy and Law.

[CR26] Gina L, Alice G, Atikah A, Richard M, Nathaniel O (2012). Moving towards universal health coverage: Health insurance reforms in nine developing countries in Africa and Asia. The Lancet.

[CR27] González P (2004). Should physicians’ dual practice be limited? An incentive approach. Health Economics.

[CR28] González P (2005). On a policy of transferring public patients to private practice. Health Economics.

[CR29] González P, Macho-Stadler I (2013). A theoretical approach to dual practice regulations in the health sector. Journal of Health Economics.

[CR30] González, P., Montes-Rojas, G., & Pal, S. (2017). Dual practice by health workers: Theory and evidence from indonesia. IZA Discussion Paper Series, 11038, Bonn, Germany: IZA.

[CR31] Gruen R, Anwar R, Begum T, Killingsworth JR, Normand C (2002). Dual job holding practitioners in Bangladesh: An exploration. Social Science & Medicine.

[CR32] Hartwig J (2008). What drives health care expenditure?—Baumol’s model of ‘unbalanced growth’ revisited. Journal of Health Economics.

[CR33] Hartwig J (2010). Is health capital formation good for long-term economic growth? - panel granger-causality evidence for oecd countries. Journal of Macroeconomics.

[CR34] Havranek T, Horvath R, Irsova Z, Rusnak M (2015). Cross-country heterogeneity in intertemporal substitution. Journal of International Economics.

[CR35] Hort K, Hipgrave DB (2013). Dual practice by doctors working in South and East Asia: A review of its origins, scope and impact, and the options for regulation. Health Policy and Planning.

[CR36] Humphrey C, Russell J (2004). Motivation and values of hospital consultants in South-East England who work in the national health service and do private practice. Social Science & Medicine.

[CR37] Kunze L (2014). Life expectancy and economic growth. Journal of Macroeconomics.

[CR38] Lim K (2017). Assessing the double-edged sword of using imitation as a stepping stone to innovation: A case of Malaysia’s k-economy puzzle. Singapore Economic Review.

[CR39] Madsen JB (2018). Health-led growth since 1800. Macroeconomic Dynamics.

[CR40] March M, Schroyen F (2005). Can a mixed health care system be desirable on equity grounds?*. The Scandinavian Journal of Economics.

[CR41] Mariani F, Pérez-Barahona A, Raffin N (2010). Life expectancy and the environment. Journal of Economic Dynamics and Control.

[CR42] McPake B, Hanson K, Adam C (2007). Two-tier charging strategies in public hospitals: Implications for intra-hospital resource allocation and equity of access to hospital services. Journal of Health Economics.

[CR43] McPake B, Hongoro C, Russo G (2011). Two-tier charging in Maputo central hospital: Costs, revenues and effects on equity of access to hospital services. BMC Health Services Research.

[CR44] McPake B, Fronteira I, Ferrinho P, Russo G (2013). Negotiating markets for health: An exploration of physicians’ engagement in dual practice in three African capital cities. Health Policy and Planning.

[CR45] McPake B, Russo G, Tseng FM (2014). How do dual practitioners divide their time? The cases of three african capital cities. Social Science & Medicine.

[CR46] Osang T, Sarkar J (2008). Endogenous mortality, human capital and economic growth. Journal of Macroeconomics.

[CR47] Pisani E, Olivier Kok M, Nugroho K (2016). Indonesia’s road to universal health coverage: A political journey. Health Policy and Planning.

[CR48] Rana RH, Alam K, Gow J (2019). Health expenditure and gross domestic product: Causality analysis by income level. International Journal of Health Economics and Management.

[CR49] Rickman N, McGuire A (1999). Regulating providers’ reimbursement in a mixed market for health care. Scottish Journal of Political Economy.

[CR50] Rokx C, Schieber G, Harimurti P, Tandon A, Somanathan A (2009). Health financing in Indonesia: A reform road map.

[CR51] Saksena, P., Xu, K., Elovainio, R., & Perrot, J. (2010). Health services utilization and out-of-pocket expenditure at public and private facilities in low-income countries. World Health Report 2010, Background Paper, 20 World Health Organization.

[CR52] Socha KZ, Bech M (2011). Physician dual practice: A review of literature. Health Policy.

[CR53] Strauss, J., Beegle, K., Sikoki, B., Dwiyanto, A., Herawati, Y., & Witoelar, F. (2004). The third wave of the indonesia family life survey: Overview and field report. WR-144/1-NIA/NICHD

[CR54] Strauss, J., Witoelar, F., Sikoki, B., & Wattie, A. (2009). The third wave of the indonesia family life survey: Overview and field report. WR-675/1-NIA/NICHD.

[CR55] Strauss, J., Witoelar, F., & Sikoki, B. (2016). The fifth wave of the indonesia family life survey: Overview and field report. WR-1143/1-NIA/NICHD.

[CR56] Thabrany, H. (2008). Politics of national health insurance of Indonesia: A new era of universal coverage. In *7th European Conference on Health Economics Rome*.

[CR57] Trimborn T, Koch KJ, Steger TM (2008). Multidimensional transitional dynamics: A simple numerical procedure. Macroeconomic Dynamics.

